# Chromosomal location of 18S and 5S rDNA sites in *Triportheus* fish species (Characiformes, Characidae)

**DOI:** 10.1590/S1415-47572009005000017

**Published:** 2009-01-23

**Authors:** Débora Diniz, Alejandro Laudicina, Luiz Antonio Carlos Bertollo

**Affiliations:** 1 Departamento de Genética e Evolução, Universidade Federal de São Carlos, São Carlos, SPBrazil; 2 Centro Nacional de Energia Atômica, Buenos AiresArgentina

**Keywords:** chromosomal markers, fish cytogenetics, NOR, ribosomal genes

## Abstract

The location of 18S and 5S rDNA sites was determined in eight species and populations of the fish genus *Triportheus* by using fluorescent *in situ* hybridization (FISH). The males and females of all species had 2n = 52 chromosomes and a ZZ/ZW sex chromosome system. A single 18S rDNA site that was roughly equivalent to an Ag-NOR was detected on the short arms of a submetacentric pair in nearly all species, and up to two additional sites were also observed in some species. In addition, another 18S rDNA cluster was identified in a distal region on the long arms of the W chromosome; this finding corroborated previous evidence that this cluster would be a shared feature amongst *Triportheus* species*.* In *T. angulatus*, a heterozygotic paracentric inversion involving the short arms of one homolog of a metacentric pair was associated with NORs. The 5S rDNA sites were located on the short arms of a single submetacentric chromosomal pair, close to the centromeres, except in *T. auritus*, which had up to ten 5S rDNA sites. The 18S and 5S rDNA sites were co-localized and adjacent on the short arms of a chromosomal pair in two populations of *T. nematurus*. Although all *Triportheus* species have a similar karyotypic macrostructure, the results of this work show that in some species ribosomal genes may serve as species-specific markers when used in conjunction with other putatively synapomorphic features.

## Introduction

The advent of fluorescent *in situ* hybridization (FISH) has made it possible to map specific DNA sequences in plants and animals. In fish, the location of ribosomal genes (45S and 5S rDNA) has been widely used to characterize species and populations and/or to establish evolutionary relationships. Two important features have contributed to the widespread use of FISH with ribosomal DNA probes, namely, the organization of rRNA genes that consist of multiple repeats and the presence of highly conserved nucleotide sequences that are very similar among eukaryotes in general.

Fish of the genus *Triportheus*, popularly known as “sardinhas de água doce”, “sardela” or “sardinha facão”, have a widespread distribution throughout South America, ranging from Colombia to Uruguay. These fish, which may reach 20-24 cm in length, are an important source of food in certain regions of Brazil such as the Amazon. In a recent review of the genus, Malabarba (2004) considered 16 valid species, with some of the names being regarded as synonyms whereas other new species were described. Among the nomenclatural changes introduced in this revision, it was included *T. elongatus* as synonymous of *T. auritus*, *T. flavus* as synonymous of *T. angulatus* and *T. paranensis* as synonymous of *T. nematurus*. This updated nomenclature was used in the present report, concerning the species now investigated as well as other species previously analyzed.

The first cytogenetic studies of *Triportheus* were done by Falcão (Falcão JN, PhD thesis, University of São Paulo, Brazil, 1988) who analyzed the species *T. albus, T. culter, T. auritus, T. angulatus* and *T.* cf. *signatus*, followed by reports for *T. guentheri,T.* cf. *auritus* and distinct populations of *T. nematurus* (Bertollo and Cavallaro, 1992; Sánchez and Jorge, 1999; Artoni *et al.*, 2001; Artoni and Bertollo, 2002; Diniz *et al.*, 2008) and *T. venezuelensis* (Nirchio *et al.*, 2007). All of these species and populations share a common diploid number, 2n = 52, and a very similar karyotypic macrostructure composed mainly of meta/submetacentric chromosomes and a ZZ/ZW sex chromosome system. The Z chromosome is the largest in the karyotype, whereas the morphology and size of the W chromosome are characteristic of each species, but always smaller than the Z element and nearly entirely heterochromatic (Falcão, *op. cit.*; Bertollo and Cavallaro, 1992; Sánchez and Jorge, 1999; Artoni *et al.*, 2001; Artoni and Bertollo, 2002; Nirchio *et al.*, 2007).

Active Ag-NORs in this fish group are usually located on the short arms of a submetacentric pair with secondary constrictions, and probably represent the main nucleolar organizer sites in *Triportheus*, although the size of the chromosomes involved may vary among species. In most species, single Ag-NORs are the common pattern, although variation in the number of these sites has been observed (Falcão, *op. cit.*; Artoni and Bertollo, 2002). *T.venezuelensis*, *e.g.*, shows marked NOR polymorphism, with up to four Ag-NORs and seven 18S rDNA sites (Nirchio *et al.*, 2007). An extra 18S rDNA site has also been reported at a terminal position on long arms of the W chromosome in *T. nematurus, T.* cf. *auritus* and *T. guentheri* (Artoni and Bertollo, 2002), and on the Z chromosome (Nirchio *et al.*, 2007). In contrast, there is no information on the chromosomal location of 5S ribosomal sites in *Triportheus* species.

In this study, we examined the chromosomal locations of the 18S and 5S rDNA sites of several *Triportheus* species and populations, and assessed the usefulness of these sites as possible chromosomal markers within and among species.

**Figure 1:**
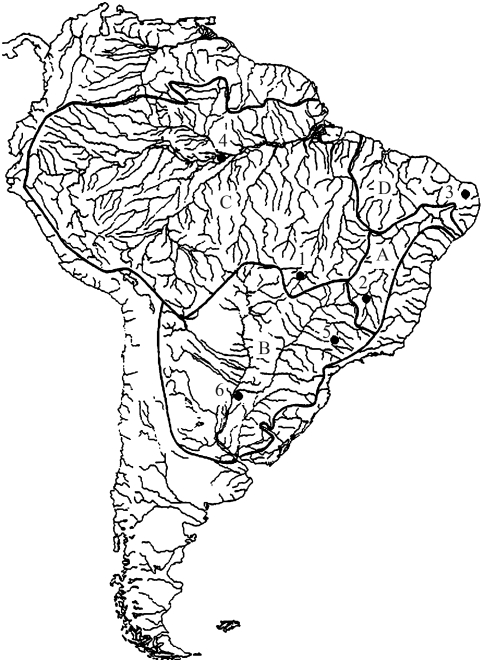
Collection sites for the *Triportheus* species analyzed. 1 = *T. trifurcatus* (Rio Araguaia, MT), 2 = *T.guentheri* (Rio São Francisco, MG), 3 = *T.* cf. *signatus* (Rio Piranha-açu, Lago Itans, RN), 4 = *T. albus*, *T. auritus*, *T. angulatus* (Rio Negro, AM), 5 = *T*. *nematurus* (Rio Piracicaba, SP), 6 = *T. nematurus* (Rio Paraná, Corrientes, Argentina).

**Figure 2:**
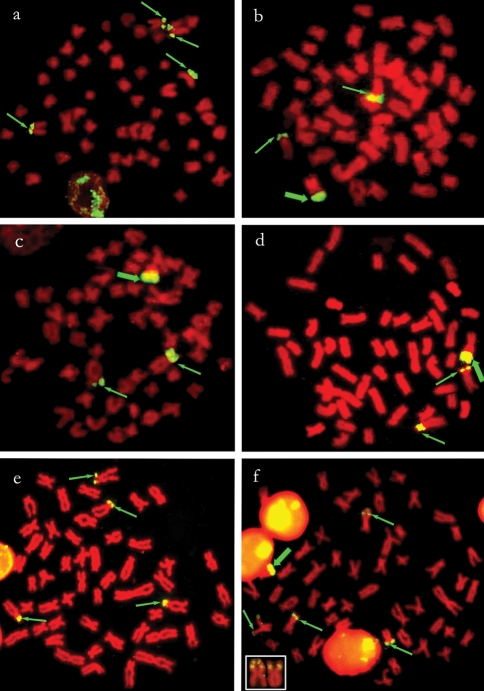
Physical map of 18S rDNA sites (arrows) in metaphase chromosomes of (a) *T. trifurcatus*, (b) *T. guentheri*, (c) *T. cf signatus*, (d) *T. albus*, (e) *T. auritus* and (f) *T. angulatus* counterstained with propidium iodide. The thick arrows indicate the location of these genes on the W chromosome. The detail in (f) shows the possible heterozygous paracentric inversion involving the NOR site.

**Figure 3:**
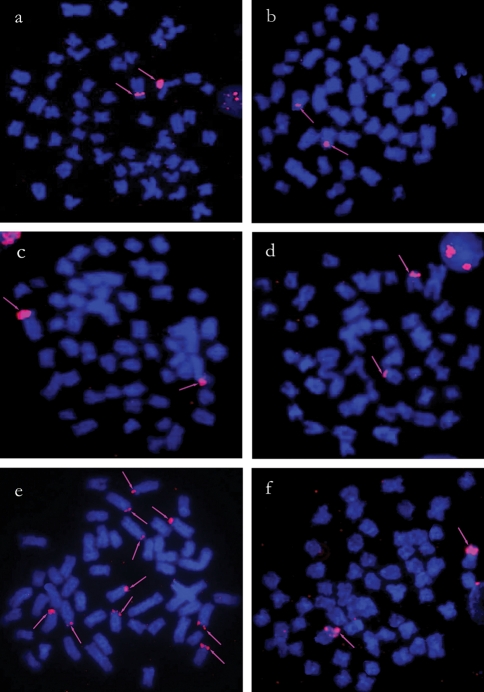
Physical map of 5S rDNA sites (arrows) in metaphase chromosomes of (a) *T. trifurcatus*, (b) *T. guentheri*, (c) *T. cf. signatus*, (d) *T. albus*, (e) *T. auritus* and (f) *T. angulatus* counterstained with DAPI.

**Figure 4:**
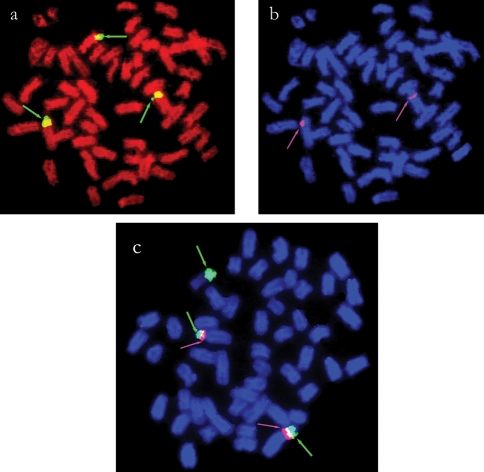
Physical map of 5S and 18S rDNA sites (arrows) showing the adjacent co-localization of both sites on chromosomes of two populations of *T. nematurus.* In (a) and (b), the 18S and 5S sites are shown, respectively, after sequential analysis in a specimen from the Rio Paraná (Corrientes, Argentina). In (c), the 18S (green signal) and 5S (magenta signal) sites were detected by double FISH in a specimen from the Rio Piracicaba (São Paulo state, Brazil). The third site bearing 18S rDNA in (a) and (c) corresponds to the NOR on the W chromosome (thick arrows). The chromosomes were counterstained with propidium iodide (a) and DAPI (b, c).

## Material and Methods

### Specimens

Eight species and populations of *Triportheus* from distinct hydrographic basins in Brazil and Argentina were analyzed (Figure 1): *T. trifurcatus* (Rio Araguaia, MT - 9 males and 5 females), *T. guentheri* (Rio São Francisco, MG - 1 male and 5 females), *T.* cf. *signatus* (Rio Piranha-açu, Lago Itans, RN - 9 males and 13 females), *T. albus* (Rio Negro, AM - 3 females), *T. auritus* (Rio Negro, AM - 7 males and 1 female), *T. angulatus* (Rio Negro, AM - 2 females), *T. nematurus* (Rio Piracicaba, SP - 23 males and 17 females), *T. nematurus* (Rio Paraná, Corrientes, Argentina - 13 males and 3 females).

### Chromosomal preparation

Mitotic chromosomes were obtained from anterior kidney cells. After killing the specimens, fragments of renal tissue were placed into 10 mL of RPMI culture medium and the cells were dissociated and processed according to Foresti *et al.* (1993).

### Fluorescent *in situ* hybridization (FISH)

The chromosomal preparations were processed for fluorescent *in situ* hybridization (FISH), as described by Pinkel *et al.* (1986), using 18S (Hatanaka and Galetti Jr., 2004) and 5S (Martins and Galetti Jr., 1999) rDNA probes. The probes were labeled by nick translation (BioNick Labeling System – Invitrogen), according to the manufacturer's instructions. Double FISH was done in *T. nematurus* to simultaneously identify 18S and 5S rDNA clusters. The stained chromosomes were analyzed by epifluorescence microscopy with an Olympus BX50 microscope and the metaphase images were captured digitally using the software CoolSNAP-pro (Media Cybernetics).

## Results

All of the species had the previously reported karyotypic features, *i.e.*, the same diploid number (2n = 52) and a similar chromosomal macrostructure consisting of meta/submetacentric chromosomes. The occurrence of a ZZ/ZW sex chromosome system was also confirmed, with the Z chromosome being the largest metacentric element in the karyotype while the W chromosome had a distinct morphology and size that varied according to the species; this chromosome was invariably smaller than the Z chromosome.

In *T. trifurcatus* and *T. auritus*, only males gave good results in FISH. In the remaining species, the results were sufficiently good to allow the location of the 18S and 5S rDNA sites in both sexes.

### 18S rDNA FISH

In *T. guentheri*, *T.* cf. *signatus*, *T. albus* and *T. nematurus*, the males and females showed two hybridization signals on the short arms of a submetacentric pair, and the females also had 18S rDNA sites at a terminal position on the long arms of the W chromosome (Figure 2b,c). Four positive sites were identified in *T. trifurcatus* and *T. auritus* and occupied the short arms of two submetacentric pairs (Figures 2a and e). In *T. angulatus*, in which only females were studied, hybridization signals were observed on five chromosomes: in two cases they were located on the short arms of a metacentric pair, in another two cases they were located on the long arms of a meta-submetacentric pair (these two sites were probably homologous despite their slight difference in size) and in one case the site was located at a distal position on the long arm of the W chromosome (Figure 2f).

### 5S rDNA FISH

Only one pair of chromosomes bore 5S rDNA in *T. trifurcatus, T. guentheri*, *T.* cf. *signatus,T. albus*, *T. angulatus* and *T. nematurus*. Although identification of the chromosomal morphology was not possible in some preparations, the 5S rDNA sites were always located on the short arms of submetacentric chromosomes, either close to centromeres or to the telomeric region (Figure 3a and 4). The only exception to this pattern was *T. auritus*, which had ten 5S rDNA sites with a similar location relative to the other species (Figure 3e). In both populations of *T. nematurus*, the 18S and 5S ribosomal genes showed adjacent co-localization on the short arms of a submetacentric chromosomal pair, as shown by sequential hybridization (Figures 4a and b) and double-labeling FISH experiments (Figure 4c). The 5S rDNA sites were located closer to centromeres than the adjacent 18S sites (Figure 4c).

## Discussion

At least two 18S rDNA sites were observed on the short arms of a submetacentric pair in virtually all of the species analyzed here. This chromosomal pair apparently corresponded to the Ag-NOR-bearing pair previously reported for *Triportheus* species (Falcão, *op. cit.*; Bertollo and Cavallaro, 1992; Artoni and Bertollo, 2002; Diniz *et al.*, 2008), showing a similar position between the silver nitrate-stained sites and the 18S hybridization signals. Two additional sites were found in *T. trifurcatus* and *T. auritus* at a similar location, but in another submetacentric pair. In *T. guentheri*, a second chromosomal pair bearing Ag-NORs was reported by Bertollo and Cavallaro (1992), but it was not detected by FISH (Artoni and Bertollo, 2002; present study). *Triportheus angulatus* also had four 18S rDNA sites located on two metacentric and two submetacentric chromosomes, in agreement with previous Ag-NOR data (Falcão, *op. cit.*). In this case, a putative paracentric inversion involving NORs may have occurred in one homologue of the metacentric pair, resulting in the subdivision of major DNA cistrons into interstitial and terminal sites on this chromosome (Figure 2f, detail).

*Triportheus* has been regarded as a karyotypically conserved fish group, based on the identical diploid number (2n = 52), similar chromosomal macrostructure and presence of a ZZ/ZW sex chromosome system in the different species. However, as shown here, this conservativeness was not applicable to nucleolus organizer regions (NORs), which varied in number, position and chromosomal location among species. In this sense, *T. venezuelensis* was also notable for the extensive polymorphism of its Ag-NORs and/or 18S rDNA sites (Nirchio *et al.*, 2007).

Another interesting chromosomal feature in *Triportheus* is the occurrence of 18S rDNA sites on the long arms of the W chromosome. This particularity has previously been reported for *T. guentheri*, *T. nematurus* (two populations) and *T.* cf. *auritus* (Artoni and Bertollo, 2002). As shown here, this feature was also found in another population of *T. nematurus*, as well as in *T.* cf. *signatus, T. albus, T. angulatus* and in a review of data for *T. guentheri*. These findings suggest that the presence of NORs on the W chromosome could be another characteristic shared by *Triportheus* species. However, in *T. venezuelensis*, 18S rDNA sites were unexpectedly located on the Z chromosome instead of the W chromosome (Nirchio *et al.*, 2007). This unusual occurrence may reflect an ancestral location of rDNA sites at equivalent regions in the Z and W chromosomes of *Triportheus*. Presumably the 18S rDNA site was eliminated from the Z chromosome of some species, while in others this site remained unchanged, although not always active.

In agreement with this hypothesis, *T. guentheri* showed sporadic Ag-NORs in the telomeric region of the long arms of Z chromosomes (Bertollo and Cavallaro, 1992), although this has not been confirmed by FISH (Artoni and Bertollo, 2002). According to Nirchio *et al.* (2007), the lack of detection of some NOR sites by FISH may reflect a reduced number of gene copies in these regions, while the corresponding Ag-NOR sites may be identified after high transcriptional gene activity. The current evidence supports the hypothesis that the NORs detected on the W chromosomes of *Triportheus* probably represent a plesiomorphic condition in this genus that most likely predates differentiation of the sex chromosome system (Artoni and Bertollo, 2002).

In all of the species studied, the 5S ribosomal genes were detected on the short arms of a single submetacentric chromosomal pair, close to centromeres. The only exception to this pattern was *T. auritus*, which had up to ten positive signals in distinct chromosomal pairs, which could be a genetic marker for this taxon. Another interesting case with a high number of 5S rDNA sites was found in *Centropyge aurantonotus* (order Percifomes), a marine reef-associated species, where up to 18 sites could be identified in the karyotype. In this case, it was suggested the probable occurrence of pseudogenes in the species' genome (Affonso and Galetti Jr., 2005).

In general, the 18S and 5S rDNA sites are located on different chromosome pairs in distinct biological groups, and this feature is also the most commonly reported situation among fish (Martins and Galetti Jr, 1999, 2000, 2001). Ribosomal cistrons consist of different multigene families composed of hundreds to thousands of copies organized in tandemly arrayed repeats. According to Dover (1986), mechanisms such as gene conversion and unequal crossovers often occur within these gene arrangements during evolution. In this context, the location of 45S and 5S rDNA sites on different chromosomes and at different positions would prevent some unfavorable rearrangements such as the translocation of 18S rDNA segments into 5S rDNA sequences and vice versa. Thus, the results for *T. nematurus*, where occurs an adjacent co-localization of 18S and 5S rDNA sites on the short arms of a single chromosomal pair, represent an unusually feature. However, it is still possible that this feature might be shared by other *Triportheus* species since 18S and 5S rDNA sites were detected on the short arms of similar chromosomes. Unfortunately, sequential hybridization or double FISH was not done in species other than *T. nematurus*. Further studies could help to establish whether this feature is a synapomorphy for this group or corresponds to a unique chromosomal marker for *T. nematurus.*
